# Analysis of deep learning-based segmentation of lymph nodes on full-dose and reduced-dose body CT

**DOI:** 10.1007/s00261-025-05253-8

**Published:** 2025-11-18

**Authors:** Lindsey H. Bloom, Tejas Sudharshan Mathai, Bohan Liu, Brandon Khoury, Naiya Patel, Olivia Wei, Praveen T. S. Balamuralikrishna, Benjamin Hou, Justin Solomon, Darko Pucar, Ehsan Samei, Elizabeth C. Jones, Ronald M. Summers

**Affiliations:** 1 Radiology and Imaging Sciences, National Institutes of Health Clinical Center, Bethesda, USA; 2 Carl E. Ravin Advanced Imaging Laboratories, Department of Radiology, Duke Medical Center, Durham, USA; 3 Walter Reed National Military Medical Center, Bethesda, USA; 4 Malcom Grow Medical Clinics & Surgery Center, JB Andrews, MD USA; 5 George Washington University Hospital, Washington, DC USA; 6 Division of Intramural Research, National Library of Medicine, National Institutes of Health, Bethesda, USA; 7 Clinical Imaging Physics Group, Department of Radiology, Duke University Health System, Durham, USA; 8 Center for Virtual Imaging Trials (CVIT), Duke University, Durham, USA

**Keywords:** CT, Lymph nodes, Reduced dose, Segmentation, Deep learning

## Abstract

**Objectives:**

The performance of fully automated deep learning-based models for the detection and segmentation of lymph nodes (LNs) on full- and simulated reduced-dose CT was validated.

**Methods:**

A total of 15,341 LNs were annotated in 151 patient CTs (age 52 ± 14 years, 87 males) from the public TCIA NIH CT Lymph Nodes dataset. Two 3D nnU-Net models were trained on 90 CT scans: (1) only full dose CTs (NoAugmentation), and (2) both full- and reduced-dose CTs (Augmentation). Dose reduction from 75% to 5% of the full-dose was simulated using a noise-addition tool. Performance was validated on the remaining 61 CTs and an external TCIA Mediastinal LNQ dataset (120 CTs, 64 females).

**Results:**

On 61 full-dose CTs, the Augmentation model detected all LNs with 67.3% precision and 84.6% sensitivity. For all LNs and large nodes (short axis diameter ≥ 8 mm), Dice Similarity Coefficient (DSC) was 0.83 ± 0.07 and 0.80 ± 0.14, while Hausdorff Distance (HD) error was 1.47 ± 0.91 mm and 3.2 ± 2.28 mm, respectively. Performance decreased with dose reduction (*p* < 0.01), reaching 73.8% detection sensitivity and 0.75 DSC at 5% dose. Statistically significant differences between Augmentation vs. NoAugmentation models were seen for all nodes (*p* < 0.001) and small nodes (*p* < 0.05) at 10% and 5% doses. On the external LNQ dataset, the Augmentation model attained a DSC of 0.76 ± 0.12 and HD of 4.7 ± 3.23 (*p* < 0.01) for all LNs.

**Conclusion:**

Degraded image quality impacted nodal delineation on reduced-dose CT. Performance improved when a model trained on both full- and reduced-dose CTs was used.

**Supplementary Information:**

The online version contains supplementary material available at 10.1007/s00261-025-05253-8.

## Introduction

The lymphatic system, comprised of lymph nodes (LNs) and vessels, plays a vital role in the body’s immune system. Lymph node enlargement (lymphadenopathy) can be an indicator of numerous pathologic conditions, including infection or metastases. Disease management is heavily dependent on the distinction between benign and malignant nodes [1, 2]. According to the tumor-node-metastasis (TNM) staging system, radiologists use nodal size, features, and expected pattern of regional nodal (N) and metastatic nodal (M) spread to assess for suspicious nodes [3]. LNs are measured with two diameters: long-axis diameter (LAD) and short-axis diameter (SAD). In current clinical practice, consensus guidelines recommend nodal measurements be done with the SAD on one axial slice to minimize measurement errors [4, 5]. However, the Lugano criteria for lymphoma assessment uses a product of the LAD and SAD [6]. Benign lymph nodes typically have SAD < 10 mm, presenting smooth borders, showing homogenous density, and containing a central fatty hilum. Conversely, suspicious nodes have an SAD greater than or equal to 10 mm, irregular borders, inhomogeneous density, and a loss of fatty hilum presentation [1, 3–5, 7]. Currently, nodal size measurement is the most important criterion for nodal involvement on anatomic imaging [4, 5]. Abnormal activity in small nodes is also seen on functional imaging with PET or SPECT, and they are often malignant in certain areas (e.g., head and neck, pelvis) for certain cancers (e.g., prostate) [8–10].

Cross-sectional imaging techniques, such as CT and MR, have become the standard for visualizing LNs [7, 11, 12]. CT is superior to MR for visualization of LNs due its spatial resolution [12] with the portal-venous phase CT (50–60 s after intravenous contrast injection) at 120 kV and full-inspiration preferred [13]. Despite this, LNs have various appearances on CT that make it challenging and tedious to delineate, while also being largely dependent on radiologist expertise. Given the importance of size measurement and single-parameter characterization of LNs as malignant or benign, this clinical standard is impractical. Moreover, many CT scans are done without intravenous contrast or at reduced doses. For example, in PET imaging, a non-contrast reduced-dose CT is acquired with quiet breathing for optimal PET co-registration. Radiologists specializing in nuclear medicine generally report only nodal activity and omit size measurements due to its cumbersome nature, which results in a loss of diagnostically valuable information.

While radiation is a powerful tool in medicine, the risk vs. benefit of radiation exposure has become an increasingly prevalent area of research. In 2006, the average radiation exposure in the US was reported to have increased from 3.6 mSv to 6.2 mSv effective dose over 20 years [14, 15]. During this time, medical radiation exposure increased from 15% to 48% of all exposure, accounting for a 3 mSv increase in effective dose per individual [15–17]. CT scans contributed to 49% of the medical exposure with a 1.47 mSv effective dose [15]. An abdomen and pelvis CT, for example, can expose patients to an effective dose of 3 to 25 mSv [15, 18]. This poses a potentially higher risk for patients with chronic disease, such as cancer, undergoing recurrent imaging studies for surveillance. While the CT exposure has been in decline [19], managing patient imaging at reduced radiation dose settings remains a priority.

A recent study [20] identified a median cumulative effective dose (CED) from recurrent imaging of 130.3 mSv over a span of 1–5 years, with a maximum CED of 1185 mSv. Such high radiation exposure is associated with an increased cancer incidence [20, 21]. As a result, reduced-dose CT has become an increasingly popular area of research. However, dose reduction increases the noise, which degrades image quality and diagnostic accuracy. For small structures, such as lymph nodes, this can pose problems, especially when measurement is paramount. Another unknown is the effect of reduced radiation doses on automated tools to delineate LNs.

The purpose of this study was to evaluate the performance of fully automated deep learning-based approaches for lymph node segmentation on CT as a function of nodal size and acquisition dose levels. Ascertaining the effect of degraded image quality on nodal detection and segmentation can provide a quantitative estimate of dose reduction for better patient management.

## Materials and methods

### Patient sample

**Fig. 1:**
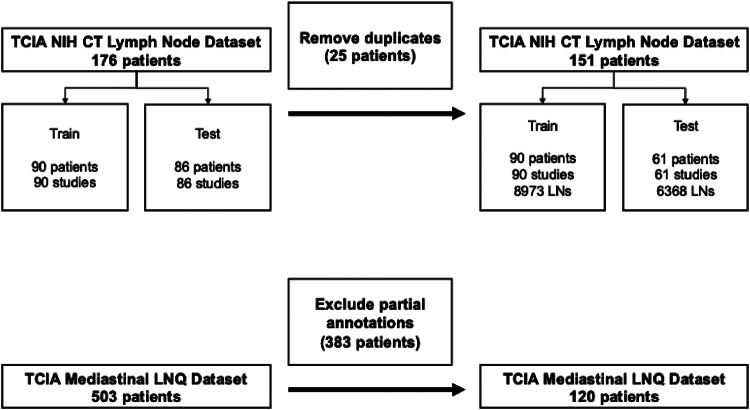
Standards for Reporting Diagnostic Accuracy (STARD) chart describing the patient sample used to evaluate two fully automated deep learning-based approaches for lymph node segmentation

This retrospective study utilized two publicly available Cancer Imaging Archive (TCIA) datasets: (1) the NIH CT Lymph Node dataset containing 176 contrast-enhanced CT volumes [22, 23] and (2) the mediastinal lymph node quantification (LNQ) dataset [24]. The study is Health Insurance Portability and Accountability Act compliant and approved by the Institutional Review Board at Institution-A. The requirement for signed informed consent from the patients was waived. Table 1 describes the patient characteristics and the CT acquisition parameters. A full description of the diverse patient disease etiologies for both datasets is provided in Supplementary Tables 1 and 2. Figure 1 summarizes the data collection procedure using a Standards for Reporting Diagnostic Accuracy (STARD) chart.

**Table 1:** Patient characteristics and CT acquisition technique for the two public TCIA datasets

		TCIA NIH CT Lymph Node Dataset			TCIA LNQ Dataset
		Total	Train	Test	
Patients		151	90	61	120
CTs		151	90	61	120
Age (years, Mean ± SD)		52 ± 14	54 ± 14	50 ± 15	–
Sex					
Male		87	55	32	56
Female		64	35	29	64
Scanners					
GE (LightSpeed Ultra)		21	14	7	–
Siemens (Definition)		74	37	37	–
Philips (Brilliance 64)		56	39	17	–
Tube Voltage Range (kVp)		[100, 120]	[100, 120]	120	–
Tube Current Range (mA)		[113, 650]	[121, 650]	[113, 649]	–
Width x Height		512 × 512	512 × 512	512 × 512	512 × 512
Num Slices (Range)		[56, 776]	[56, 776]	[411, 755]	[48, 353]
Voxel Spacing Range (x, y, z)		0.6–1, 0.6–1, 1–5	0.6–1, 0.6–1, 1–5	0.6–1, 0.6–1, 1–5	0.6–1, 0.6–1, 1.25–5
Body Region					
Chest-Abdomen-Pelvis		143	87	56	0
Chest		1	1	0	120
Chest-Abdomen		1	1	0	0
Abdomen-Pelvis		6	1	5	0

Supplementary Table 1 describes the various disease etiologies for the patients in the public TCIA NIH CT Lymph Node dataset. Briefly, the patients had lymphadenopathy arising from various disease etiologies, such as melanoma, chronic lymphocytic leukemia (CLL), lymphoma, mesothelioma, cancers of the thyroid, lung, thymus, breast, esophagus, liver, pancreas, kidneys, colon, ovaries, and among others. Identification and removal of 25 duplicate volumes resulted in a total of 151 full-dose CT volumes for this study. Of these, 90 volumes (filenames starting with “MED”) were used for training, including 87 chest-abdomen-pelvis scans, 1 chest-abdomen, 1 abdomen-pelvis, and 1 chest scan. The remaining 61 volumes (filenames starting with “ABD”) were reserved for testing and consisted of 56 chest-abdomen-pelvis scans and 5 abdomen-pelvis scans.

The external public TCIA mediastinal LNQ dataset contained 513 patients and came from multiple institutions (Massachusetts General Hospital, Dana-Farber Cancer Institute, Brigham and Women’s Hospital). Partial annotations of lymph nodes were provided for 393 patients, while 120 patients had all mediastinal lymph nodes fully annotated. The fully annotated data subset was selected for analysis in this work. Supplementary Table 2 provides full details on the diverse patient disease characteristics for this data subset. Briefly, it included those patients with non-small cell lung cancer (NSCLC), CLL, lymphoma, and various other cancer types.

### Reference standard

Segmentation masks were provided with the TCIA NIH CT Lymph Node dataset for LNs that were considered enlarged and clinically significant (SAD ≥ 10 mm) [22, 23]. However, upon thorough review, several LNs with SAD ≥ 10 mm were not annotated in this dataset. Additionally, in many instances, masses were annotated instead of nodes.

To obtain a reliable reference standard, two board-certified radiologists (30 + years of experience) and four radiology residents (2 + years of experience) were recruited to identify LNs of all sizes in the 151 full-dose CT volumes. The residents initially annotated 8973 LNs in the 90 full-dose CTs (average of 99 LNs per volume). The testing data subset of 61 CTs was verified entirely by the two board-certified radiologists (30 + years of experience). The first radiologist reviewed the resident annotations and identified the center point (3D-coordinate) of nodes that were either missed or incorrectly annotated. Next, a research fellow (2 + years of experience) manually corrected the annotations. Finally, the second radiologist independently reviewed the nodes identified by the first radiologist and annotations performed by the fellow. If the second radiologist spotted annotation errors or any nodes that were missed, their centers were marked, and the same research fellow corrected them. For example, annotations made incorrectly in the pericardial recess were erased. A total of 6368 LNs were identified in the 61 volumes by the senior radiologists.

The full dataset contained 15,341 LNs of all sizes. To account for variability in measurements by radiologists, this work considered LNs with SAD ≥ 8 mm to be large and nodes with SAD ≥ 3 mm but below 8 mm to be small. Nodes with SAD < 3 mm were not considered as they were less likely to be of clinical significance. The training dataset contained 2139 large nodes and 5966 small nodes (868 nodes with SAD < 3 mm were ignored). The test dataset contained 800 large nodes and 4078 small nodes (1490 nodes with SAD < 3 mm were ignored).

### Generation of reduced-dose CT

**Fig. 2:**
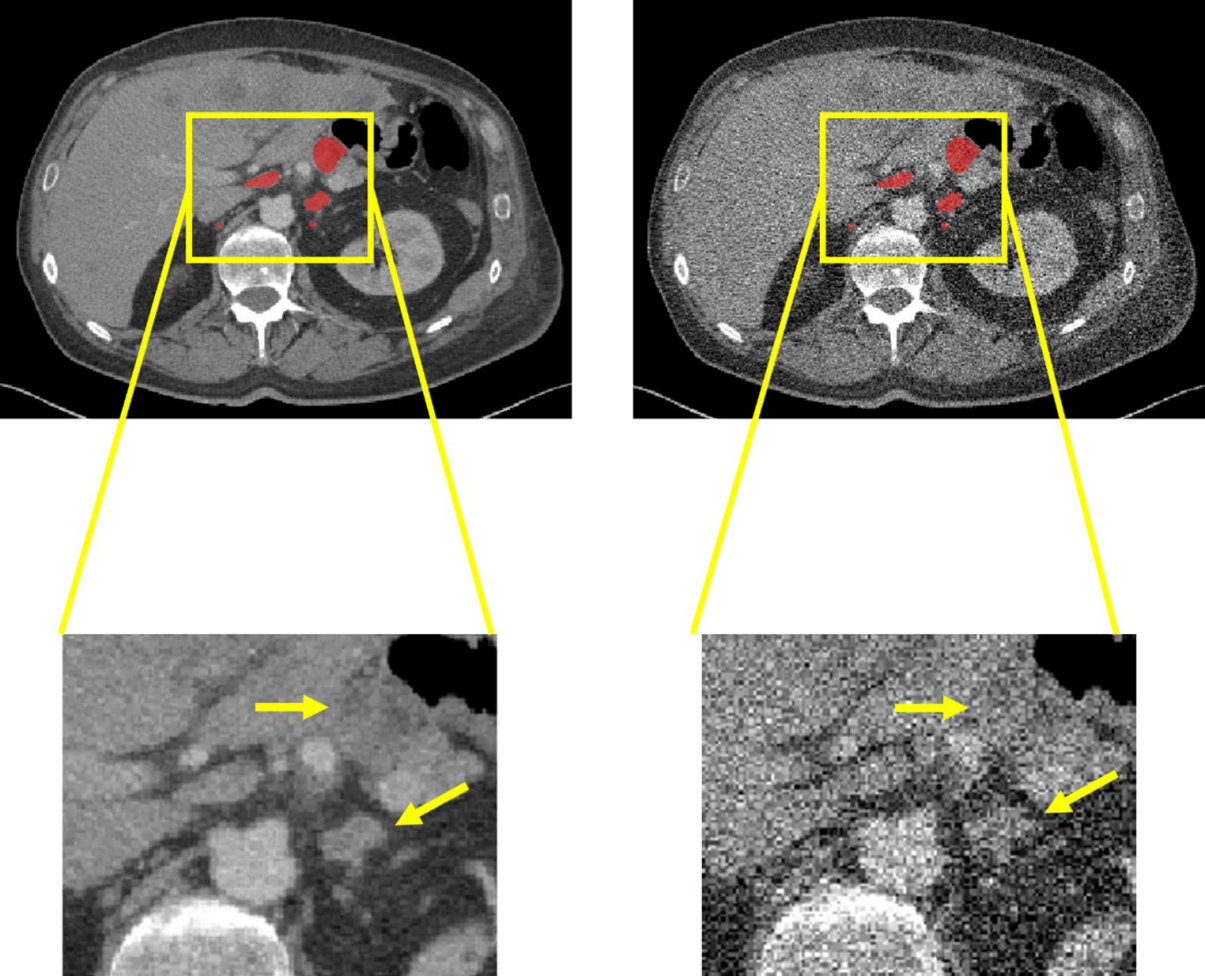
Visual example of degraded image quality and lymph node (red) visualization on a full dose CT (left) vs. 10% dose CT (right). The yellow arrows highlight two enlarged lymph nodes in the retroperitoneum of a male patient (age 61 years) with short axis diameter (SAD) of 2.3 cm and 1.7 cm, respectively. Two smaller lymph nodes are not clearly visible in the reduced dose CT

Reduced-dose CTs were simulated with a previously validated image-domain noise-addition tool [25]. The tool injected synthetic noise into the standard dose CT by estimating and replicating the magnitude, texture, and non-stationary properties of noise observed in reduced-dose CT acquisitions. Specifically, it performed forward projection of the CT image data, white noise generation in the sinogram domain, filtering based on the image-specific noise power spectrum, followed by back-projection and dose-dependent scaling of the noise prior to its addition to the image. The intent behind the use of reduced-dose CT was to leverage a 3D lymph node segmentation model trained only on full-dose CT and evaluate its performance on reduced-dose CT, where the quality has been degraded. Figure 2 shows an example of the degraded quality of the reduced-dose CT in comparison to the original full-dose.

For each of the 61 full-dose testing volumes, five corresponding reduced-dose CTs were generated at 75%, 50%, 25%, 10%, and 5% dose settings. As a result, including the full-dose CT, a total of 366 CT volumes were available for testing (61 CTs at each dose). As the anatomy did not change when simulating a reduced-dose CT, the segmentation mask from the full-dose CT was directly transferred to the reduced-dose CT.

### Deep learning model

**Fig. 3:**
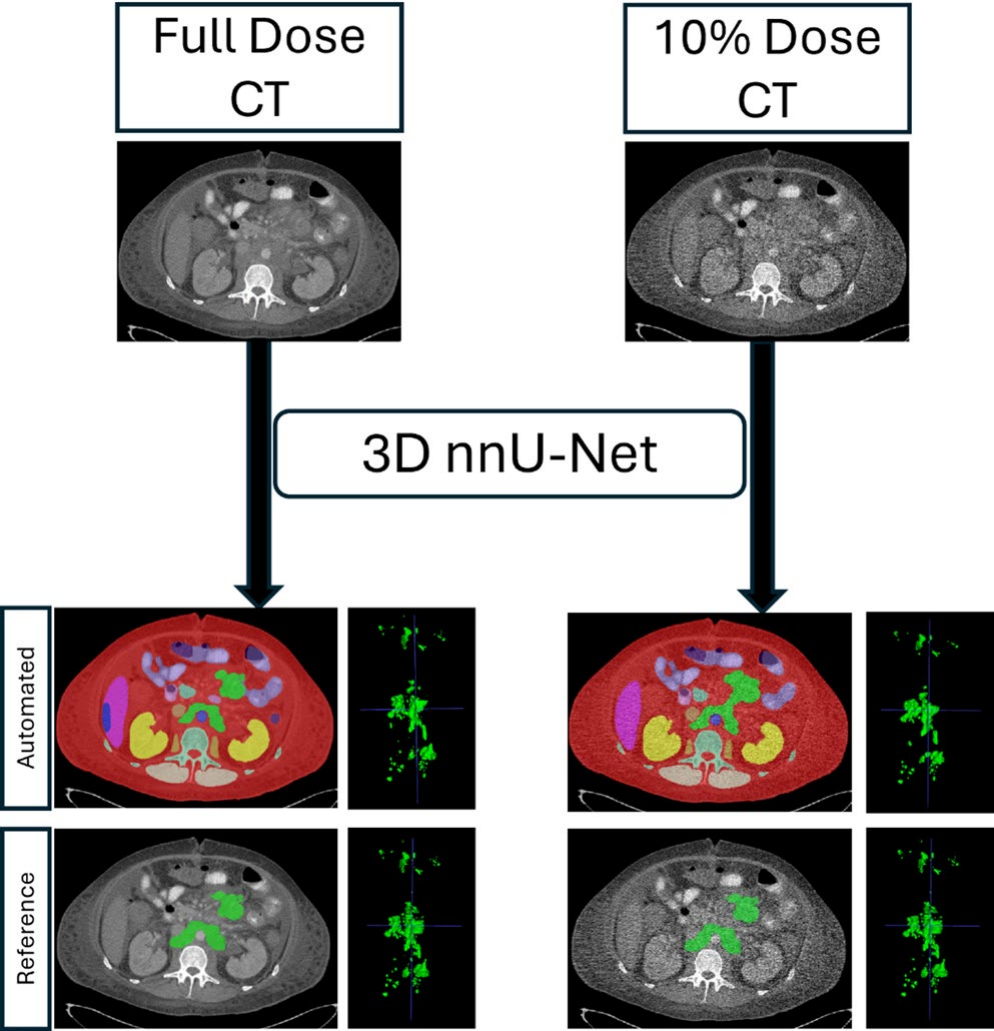
Framework for the detection and segmentation of lymph nodes on full- and reduced-dose body CT. The input CT volume (full or reduced-dose) was fed to a 3D full-resolution nnU-Net that segmented the lymph nodes (green) along with other anatomical structures (various colors). The reference annotation in 2D as well as in 3D is shown for comparison

Figure 3 shows the overall framework. A previously validated deep learning-based 3D model [26] trained with the 90 full-dose CTs was used to segment LNs. We refer to this model as the “NoAugmentation” model henceforth. The model was built with the nnU-Net framework [27], which is regarded as the de facto standard for segmentation tasks [27] due to its superior performance on many tasks, such as multi-organ segmentation in CT and MRI among others [27]. It has often outperformed other architectures, such as transformer-based approaches [28]. The 3D full-resolution nnU-Net model segmented 29 different structures, which included LNs and other anatomical structures (e.g., liver, pancreas, skeleton). As LNs straddle major organs, the anatomical priors were used to reduce the number of false positives by distinguishing nodes from other adjacent structures. At test time, only the predicted LNs were retained while the remaining 28 classes were discarded.

### Comparison

Another 3D nnU-Net model was trained on an augmented dataset comprising of both the full-dose and reduced-dose simulations. The augmented dataset had 540 CT volumes including both the full-dose and reduced-dose CTs generated at 75%, 50%, 25%, 10%, and 5% dose settings (90 CTs at each dose). We refer to this model as the “Augmentation” model henceforth. The performance of the Augmentation model was compared against the NoAugmentation model described above.

### Statistical analysis

Detection performance was evaluated using precision (or positive predictive value), sensitivity (or recall), and F1-score (harmonic mean of precision and sensitivity). Segmentation performance was quantified with Dice Similarity Coefficient (DSC) and Hausdorff Distance (HD) error (in mm). Dice score measures the overlap between the reference annotation and the prediction, while HD error estimates the distance between the two. Higher Dice scores and lower HD errors indicate good performance. Metrics were calculated for three groups: (1) all LNs, (2) large nodes with SAD ≥ 8 mm, and (3) small nodes with SAD ≥ 3 mm but below 8 mm. Model performance at different doses were statistically evaluated using one-way repeated measures Analysis of Variance (ANOVA) and post-hoc tests that adjusted (Bonferroni) for multiple comparisons (“anova_test” and “wilcox_test” functions, “rstatix” package, RStudio v. 2024.04.2 + 764). A *p*-value < 0.05 was considered statistically significant.

## Results

### Patient characteristics

The TCIA NIH CT Lymph Node dataset contained 151 patients (age: 52 [mean] ± 14 [SD] years; 87 males). The TCIA mediastinal LNQ dataset contained 120 patients (age: unknown, 64 females).

### Detection performance

Table 2 summarizes the detection performance. For the TCIA NIH CT Lymph Node dataset, on full-dose CT, the NoAugmentation model detected LNs of all sizes with a 70.7% precision, 83.8% sensitivity, and 76.8% F1-score. As the dose was reduced, the precision increased for the NoAugmentation model while the sensitivity and F1-score decreased. Sensitivity and F1-score dropped to 60.0% and 68.3% at a 5% dose where the model missed 1951 LNs (~ 40% of all nodes). Conversely, the number of false positives dropped to 766 at 5% dose in contrast to 1,692 at a full dose. Precision, sensitivity and F1-score for detecting LNs were above 75% until the dose dropped below 25%. As shown in Supplementary Fig. 1, a logarithmic relationship was observed between dose and precision (R^2^ = 0.98), sensitivity (R^2^ = 0.95), and F1-score (R^2^ = 0.9).

**Table 2:** Metrics for automated detection of lymph nodes on CT

Dose	Augment	TP	FP	FN	PPV (Precision)	Sensitivity	F1-score
TCIA NIH CT Lymph Nodes Dataset (61 volumes)							
Full	No	4092	1692	786	**70.7**	83.8	**76.8**
	Yes	4129	2010	749	67.3	**84.6**	74.9
75%	No	4073	1628	805	**71.4**	83.5	**77.0**
	Yes	4122	1977	756	67.6	**84.5**	75.1
50%	No	4027	1513	851	**72.7**	82.6	**77.3**
	Yes	4111	1893	767	68.5	**84.3**	75.6
25%	No	3858	1299	1020	**74.8**	79.1	**76.9**
	Yes	4056	1769	822	69.6	**83.1**	75.8
10%	No	3432	980	1446	**77.8**	70.4	73.9
	Yes	3854	1507	1024	71.9	**79.0**	**75.3**
5%	No	2927	766	1951	**79.3**	60.0	68.3
	Yes	3600	1198	1278	75.0	**73.8**	**74.4**
TCIA Mediastinal LNQ Dataset (120 volumes)							
Full	No	777	651	190	**54.4**	80.4	**64.9**
	Yes	789	694	178	53.2	**81.6**	64.4

Bold font indicates best results

Precision, sensitivity, and F1-score are described in percentages

A total of 4878 lymph nodes were annotated by two expert radiologists in 61 CTs in the TCIA Lymph Nodes dataset, while 967 lymph nodes (above 3 mm) were present in the TCIA LNQ dataset

In contrast, the Augmentation model obtained 67.3% precision, 84.6% sensitivity, and 74.9% F1-score at full dose. At lower doses, the precision increased while the sensitivity and F1-score decreased. However, compared to the NoAugmentation model, this model showed marked improvements in nodal detection with higher sensitivity and F1-score at 10% and 5% doses. At a 5% dose, the model only missed 1278 LNs (~ 26% of all nodes vs. ~40% for NoAugmentation). Despite this improvement in sensitivity, the Augmentation model had a lower precision of 75% at a 5% dose due to more false positives.

On the TCIA mediastinal LNQ dataset, the performance difference between the Augmentation and NoAugmentation models was less than 2%. The Augmentation model obtained 53.2% precision, 81.6% sensitivity, and 64.4% F1-score, whereas the NoAugmentation model obtained 54.4% precision, 80.4% sensitivity, and 64.9% F1-score. Sensitivity was higher for the Augmentation model with fewer false negatives.

### Segmentation performance

**Fig. 4:**
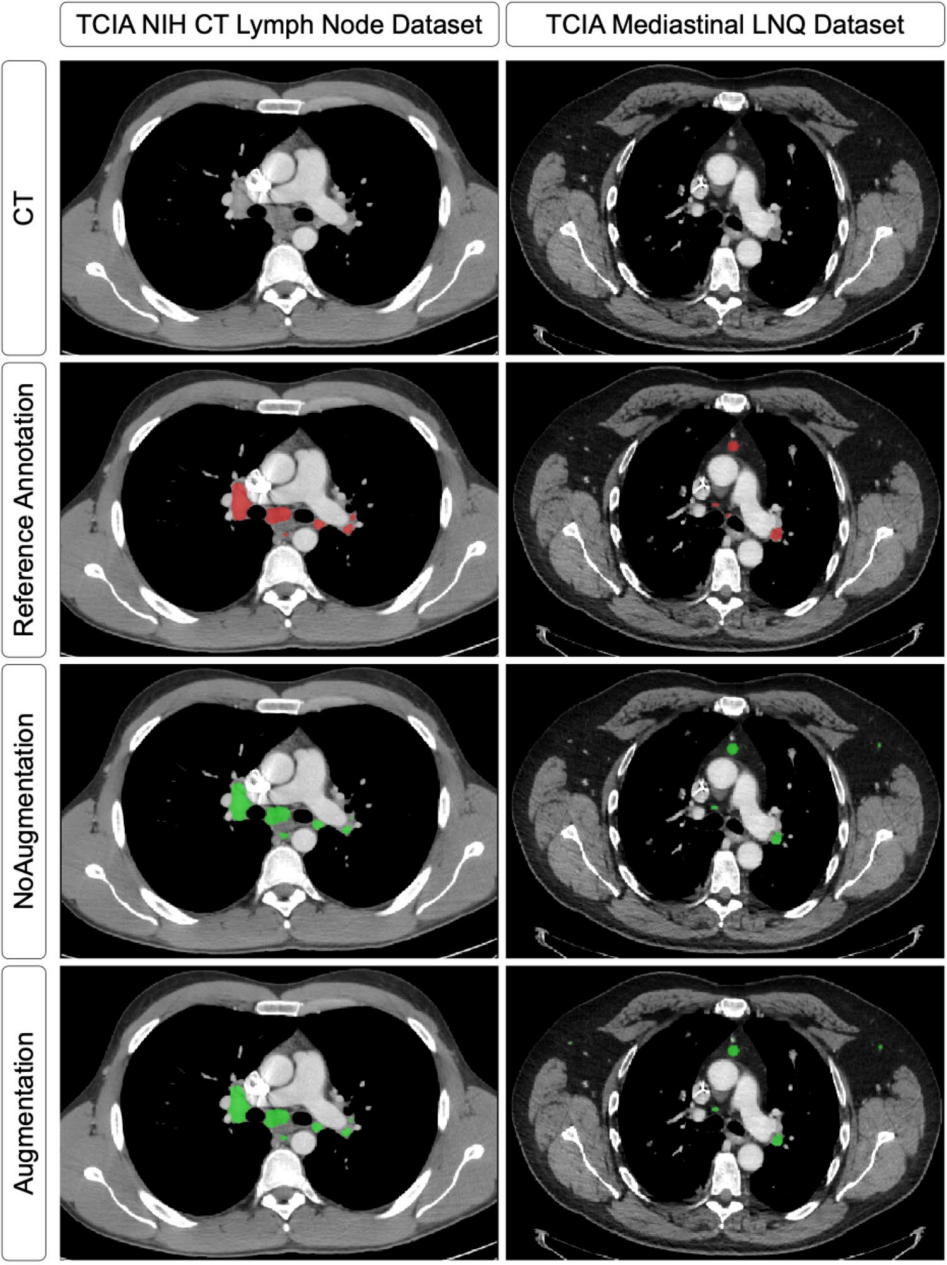
Segmentation results for large and small lymph nodes by the NoAugmentation 3D nnU-Net model (only full dose CTs) and the Augmentation model (both full- and reduced-dose). Left column shows a male patient from the TCIA NIH CT Lymph Node dataset with chronic lymphocytic leukemia (CLL). Note the bulky lymph node conglomeration in the right hilum. The right column shows a male patient with colorectal cancer in the external TCIA Mediastinal LNQ dataset. Both models identified large nodes relatively well, but they missed a small node at the carina

**Fig. 5:**
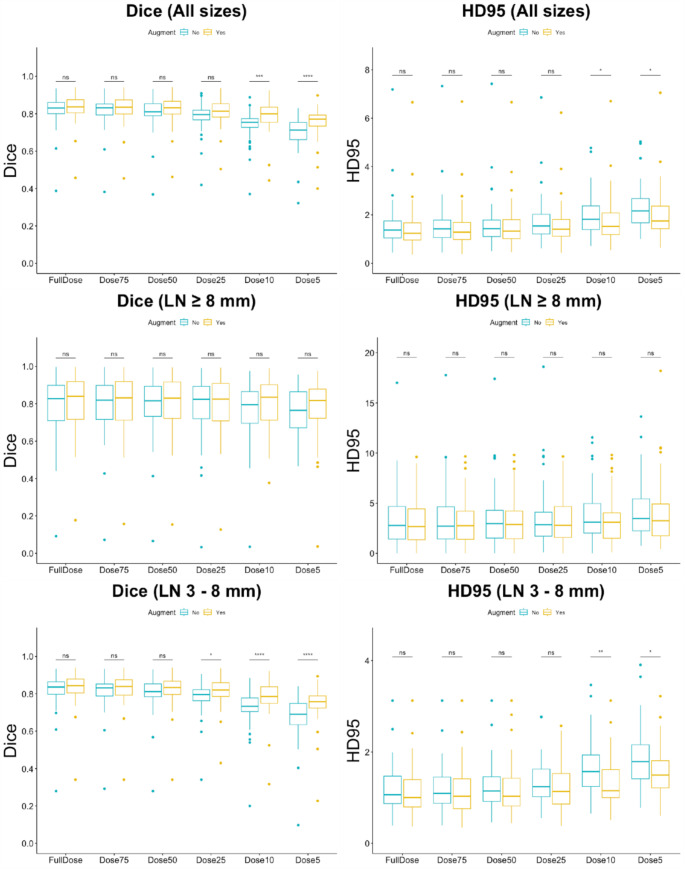
For the TCIA NIH CT Lymph Nodes dataset (61 volumes), box plots show the Dice scores and Hausdorff Distance (HD) errors (in mm) for segmentation of lymph nodes of different sizes. Results are shown for each dose level. A model trained solely on full-dose CTs is compared against a different model trained with both full- and reduced-dose CTs. “*” indicates a p-value less than 0.05

Table 3 details the segmentation performance for both datasets, while Fig. 4 shows qualitative examples of nodal segmentations. For the TCIA NIH CT Lymph Node dataset, Fig. 5 shows the distribution of Dice scores and HD errors for all, large, and small LNs, respectively. On full-dose CT, the NoAugmentation model segmented all LNs with a DSC of 0.82 ± 0.08 and an HD error of 1.54 ± 0.94 mm, large nodes with a DSC of 0.79 ± 0.15 and HD error of 3.36 ± 2.69 mm, and small nodes with a DSC of 0.82 ± 0.08 and HD of 1.17 ± 0.49 mm.

**Table 3:** Metrics for automated segmentation of lymph nodes on CT

Dose	Augment	All sizes		SAD ≥ 8 mm		3 mm ≤ SAD < 8 mm	
		DSC	HD95 (mm)	DSC	HD95 (mm)	DSC	HD95 (mm)
TCIA NIH CT Lymph Nodes Dataset (61 volumes)							
Full	No	0.82 ± 0.08	1.54 ± 0.94	0.79 ± 0.15	3.36 ± 2.69	0.82 ± 0.08	1.17 ± 0.49
	Yes	**0.83 ± 0.07**	**1.47 ± 0.91**	**0.80 ± 0.14**	**3.20 ± 2.28**	**0.83 ± 0.08**	**1.12 ± 0.51**
75%	No	0.81 ± 0.07	1.58 ± 0.95	0.79 ± 0.15	3.52 ± 2.88	0.81 ± 0.08	1.20 ± 0.47
	Yes	**0.82 ± 0.07**	**1.48 ± 0.92**	**0.80 ± 0.14**	**3.21 ± 2.28**	**0.82 ± 0.08**	**1.13 ± 0.52**
50%	No	0.80 ± 0.08	1.62 ± 0.96	0.79 ± 0.15	3.48 ± 2.85	0.80 ± 0.08	1.24 ± 0.47
	Yes	**0.82 ± 0.07**	**1.53 ± 0.91**	**0.80 ± 0.14**	**3.29 ± 2.32**	**0.82 ± 0.08**	**1.17 ± 0.54**
25%	No	0.78 ± 0.07	1.75 ± 0.91	0.79 ± 0.16	3.66 ± 3.05	0.78 ± 0.08	1.36 ± 0.46
	Yes	**0.81 ± 0.06**	**1.57 ± 0.85**	**0.79 ± 0.14**	**3.34 ± 2.26**	**0.81 ± 0.07**	**1.20 ± 0.46**
10%	No	0.74 ± 0.07	2.01 ± 0.79	0.76 ± 0.15	3.80 ± 2.48	0.72 ± 0.09	1.63 ± 0.54
	Yes	**0.78 ± 0.07**	**1.72 ± 0.91**	**0.79 ± 0.13**	**3.35 ± 2.23**	**0.78 ± 0.08**	**1.36 ± 0.50**
5%	No	0.70 ± 0.08	2.30 ± 0.87	0.76 ± 0.12	4.11 ± 2.60	0.68 ± 0.11	1.82 ± 0.58
	Yes	**0.75 ± 0.07**	**1.97 ± 0.90**	**0.77 ± 0.16**	**3.85 ± 3.04**	**0.74 ± 0.09**	**1.56 ± 0.47**
TCIA Mediastinal LNQ Dataset (120 volumes)							
Full	No	0.75 ± 0.12	4.83 ± 3.04	0.81 ± 0.11	5.17 ± 3.67	0.65 ± 0.21	4.26 ± 2.76
	Yes	**0.76 ± 0.12**	**4.7 ± 3.23**	**0.83 ± 0.12**	**5.06 ± 2.24**	**0.67 ± 0.21**	**3.94 ± 2.74**

Bold font indicates best results

“No” augmentation stands for the model trained with only the full dose CT scans, while “Yes” stands for the model trained with both the full- and reduced-dose CT scans

As the dose was reduced, DSC for the NoAugmentation model decreased across all three size groups and significant differences in segmentation performance were observed compared to the reference full-dose CT (all sizes: *p* < 0.01, large nodes: *p* < 0.05, and small nodes: *p* < 0.05). A corresponding increase in HD error for all sizes (*p* < 0.05) and large nodes (*p* < 0.05) was seen. Small nodes followed a similar trend with HD error increasing (*p* < 0.05), except between full and 75% dose (*p* > 0.05).

Compared against the NoAugmentation model, the Augmentation model obtained higher DSC and lower HD errors across all sizes of LNs. However, significant differences were only observed between the two models at 10% and 5% dose levels for all LNs (*p* < 0.05) and small nodes (*p* < 0.05). Across all doses, no difference was seen between the two models for large nodes. Comparing the full dose performance to other doses, the Augmentation model showed significant differences (*p* < 0.05), except for the 75% dose (*p* > 0.05).

**Fig. 6:**
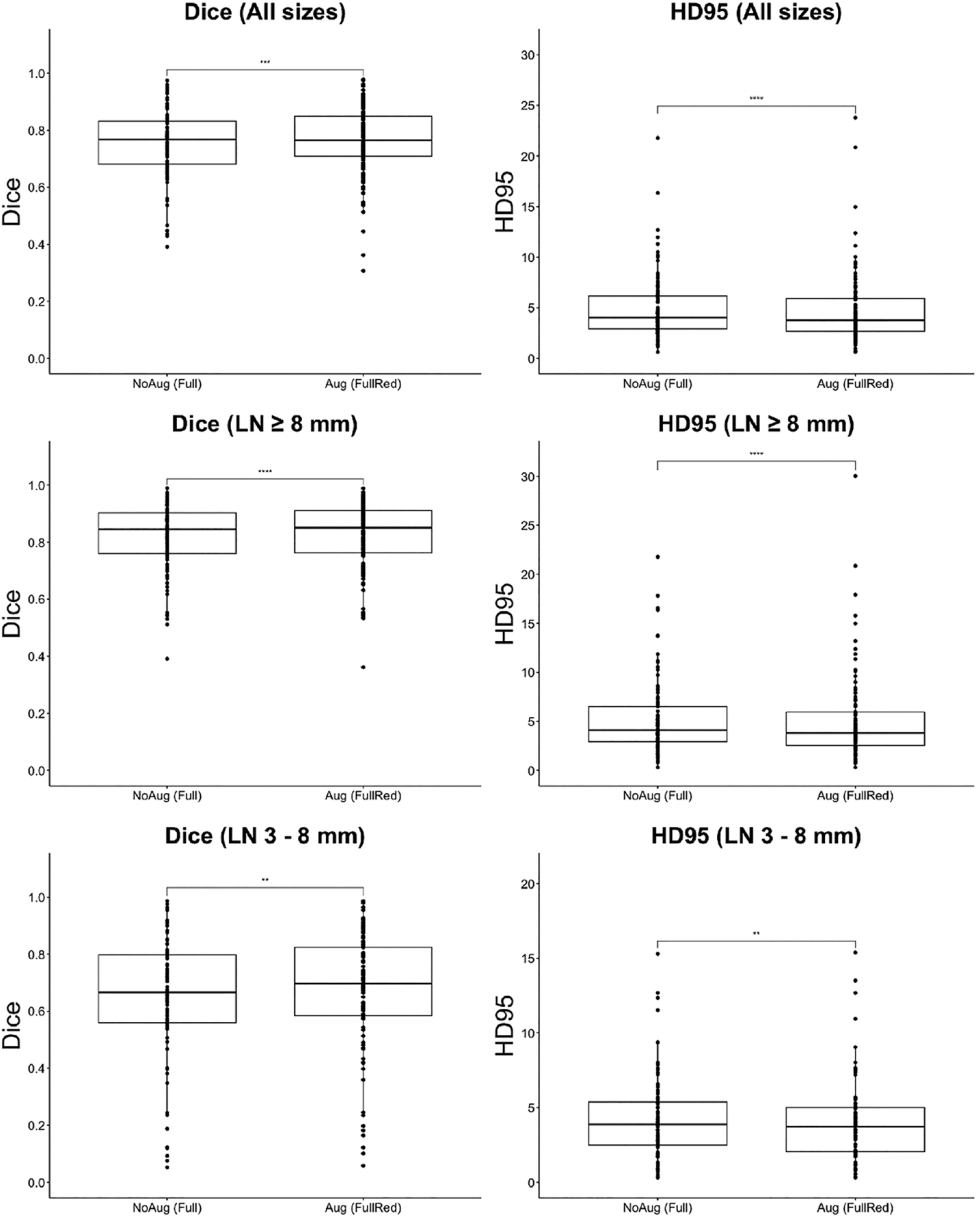
For the TCIA Mediastinal LNQ dataset (120 volumes), box plots show the Dice scores and Hausdorff Distance (HD) errors (in mm) for segmentation of lymph nodes of different sizes. These results compare a model trained solely on full-dose CTs against a different model trained with both full- and reduced-dose CTs. “*” indicates a p-value less than 0.05

For the TCIA mediastinal LNQ dataset, Fig. 6 shows the distribution of Dice scores and HD errors for all, large, and small LNs, respectively. Significant differences were observed with the Augmentation model as it segmented all LNs with a DSC of 0.76 ± 0.12 (*p* < 0.001) and an HD error of 4.7 ± 3.23 mm (*p* < 0.001), large nodes with a DSC of 0.83 ± 0.12 (*p* < 0.001) and HD error of 5.06 ± 2.24 mm (*p* < 0.001), and small nodes with a DSC of 0.67 ± 0.21 (*p* < 0.01) and HD of 3.94 ± 2.74 mm (*p* < 0.01).

## Discussion

This study quantified the effect of degraded CT image quality on the identification and delineation of lymph nodes on CT. The segmentation performance of LNs on two TCIA datasets was determined using two 3D nnU-Net models. The first model (“NoAugmentation”) was trained only on full-dose CT scans, while the second model (“Augmentation”) was trained on both full- and reduced-dose CTs. The ability of both models to generalize to simulated reduced-dose CT scans and an external public dataset (TCIA mediastinal LNQ dataset) was determined.

Results indicated that both models performed well on full-dose CT, but their performance deteriorated as dose was reduced. Detection sensitivity for the NoAugmentation model declined with reduced dose, especially below the 25% dose threshold, reflecting the expected challenges in identifying small structures on reduced-dose CT. A logarithmic relationship was observed between dose and detection metrics, which suggests that the NoAugmentation model’s performance degraded in a quantifiable manner. In contrast, the Augmentation model showed higher nodal detection sensitivity and F1-score at 10% and 5% doses. This is very important for PET/CT, which primarily utilizes low dose (25–70%) non-contrast CT, as many small metabolically active and suspicious nodes often need to be detected.

Segmentation metrics for the Augmentation model indicated that the DSC for all LNs were above 0.75 across all doses, reaching 0.82 at 75% dose and a maximum of 0.83 at full dose. DSC was largely stable for large nodes up to a 10% dose reduction (compared to 25% dose for NoAugmentation). The Augmentation model also detected and segmented small nodes relatively well, which indicated model generalizability. An expected decrease in performance was observed below 25% dose. Despite this, our findings highlight the robustness of both models in the presence of degraded CT quality, with the Augmentation model faring better for all LNs (*p* < 0.001) and small nodes (*p* < 0.001) at 10% and 5% doses.

The NoAugmentation model used in this work previously achieved 0.68 DSC for large mediastinal LNs (SAD ≥ 8 mm) [26] on the external public dataset from St. Olavs Hospital in Trondheim, Norway [29]. On the external TCIA mediastinal LNQ dataset, both models achieved similar detection performance. However, the Augmentation model achieved higher DSC and lower HD errors for all LNs (*p* < 0.001), large nodes (*p* < 0.001), and small nodes (*p* < 0.01). To our knowledge, these results represent the best performance on the TCIA LNQ dataset [24].

Our study has several key strengths. First, our lymph node dataset contains over 15,000 separate nodes, and to our knowledge, it is the largest to date. Historically, automated lymph node segmentation has been a difficult endeavor due to the small size, varying imaging characteristics, and lack of radiologist-verified reference annotations. Second, the use of anatomy prior-guided training enabled the model to better detect and distinguish lymph nodes from adjacent structures [26]. Third, the high-quality labels used for training the 3D segmentation model with full supervision was critical to achieve good segmentation results. Fourth, the models used in this work were evaluated on an external TCIA mediastinal LNQ dataset and showed their capabilities for translation to unseen patient cohorts. Lastly, the performance of the Augmentation model on reduced-dose CT suggests its direct clinical relevance for patients undergoing repeated imaging for chronic disease where cumulative radiation exposure may pose extra risk. While these patients can benefit from less radiation dose, reducing the dose degrades image quality and can notably impact the usefulness of the exam.

There are several limitations to this study. First, the test data subset used in this work was fully verified by two board-certified radiologists (30 + years of experience) and took ~ 2 months for 61 CT volumes. The training dataset was annotated by four residents, which took ~ 3 months to complete. Annotations are heavily dependent on radiologist expertise and difficult to generate at scale (> 10,000 patients) because it is cumbersome to identify and label more than 100 nodes on body CT. Additionally, there may be inter-observer variabilities in annotation, which again cannot be evaluated at scale due to the tedious nature of annotation and verification. This further necessitates the need for a fully automated tool to delineate LNs on CT. Second, the technique used in this work to simulate a dose-reduced CT introduced noise artificially and may not always fully or realistically replicate all aspects of reduced-dose image acquisition [25], such as changes in contrast dynamics on diagnostic reduced-dose CT or complete omission of contrast and quiet breathing (instead of breath-hold) acquisition on PET/CT. Third, only a 3D full-resolution nnU-Net was used in this work, and no modifications were made to the model. While other deep learning models can be used, such as transformers, the difference between such models and nnU-Net for multi-structure segmentation was less than 1% [28]. Fourth, CT scans were not intrinsically acquired with reduced dose and only simulated in this work. Incorporating such reduced dose CTs would benefit model training, while also enabling correlation with human interpretation. Lastly, benign and suspicious lymph nodes may exhibit different imaging attributes on CT, such as inhomogeneous density and presence of a fatty hilum. These differences could influence the segmentation capabilities of the models. This aspect was not addressed in the present study and will be evaluated in future work.

In summary, degraded image quality impacted the segmentation of LNs on reduced-dose CT scans. Our findings suggest that fully automated tools for segmentation of lymph nodes can largely retain their performance at 25% dose reduction. Maintaining detection sensitivity and segmentation accuracy at substantially reduced doses required model training to be optimized with the inclusion of reduced-dose CT.

## Supplementary Information

Below is the link to the electronic supplementary material.

Supplementary Material 1

## Data Availability

The study uses the publicly available TCIA Lymph Node dataset (https://www.cancerimagingarchive.net/collection/ct-lymph-nodes/).It also uses the TCIA Mediastinal LNQ dataset (https://www.cancerimagingarchive.net/collection/mediastinal-lymph-node-seg/).
