# Supplementary material for: Analysis of deep learning-based segmentation of lymph nodes on full-dose and reduced-dose body CT
Source: Abdom Radiol (NY). 2025 Nov 18;51(6):3190–201. doi: 10.1007/s00261-025-05253-8 (PMC13109288; doi:10.1007/s00261-025-05253-8)
Supplement: Supplementary file 1 — Supplementary Material 1 [file 261_2025_5253_MOESM1_ESM.docx]

# **Analysis of Deep Learning-Based Segmentation of Lymph Nodes on Full-Dose and Reduced-Dose Body CT**

**Supplementary Material**

Supplementary Table 1. Distribution of disease etiologies for the TCIA Lymph Node dataset.

| **Clinical Indication** | **Count** |
| --- | --- |
| Melanoma | 24 |
| Chronic Lymphocytic Leukemia (CLL) | 14 |
| Colorectal Cancer | 9 |
| Ovarian Cancer | 8 |
| Mesothelioma | 8 |
| Diffuse Large B-cell Lymphoma | 6 |
| Lung Cancer | 6 |
| Lung Cancer (Non-Small) | 5 |
| Adrenocortical Cancer | 5 |
| Breast Cancer | 4 |
| Renal Cancer | 4 |
| HIV | 4 |
| Thyroid Cancer | 3 |
| Prostate Cancer | 3 |
| Lymphangioleiomyomatosis (LAM) | 3 |
| HIV | 3 |
| Thymic Cancer | 2 |
| Pheochromocytoma | 2 |
| Desmoplastic Small Round Cell Tumor | 2 |
| Chronic Granulomatous Disease (CGD) | 2 |
| Autoimmune Lymphoproliferative Syndrome (ALPS) | 2 |
| Hairy Cell Leukemia (HCL) | 2 |
| Non-Hodgkin's Lymphoma | 2 |
| Hodgkin's Lymphoma | 2 |
| Mantle Cell Lymphoma | 2 |
| Primary Effusion Lymphoma (w/ Kaposi's Sarcoma) | 1 |
| Lymphoma (unknown) | 1 |
| Anaplastic Large Cell Lymphoma (ALCL) | 1 |
| x-linked agammaglobulinemia (XLA) | 1 |
| Von-Hippel Lindau (VHL) Disease | 1 |
| Neuroendocrine Tumor (NET) | 1 |
| Carcinoid Tumor | 1 |
| Myositis (w/ Hypothyroidism) | 1 |
| MEN2B (w/ medullary thyroid cancer) | 1 |
| Multicentric Castleman Disease (MCD) | 1 |
| Job's Syndrome | 1 |
| Graft-versus-Host Disease (GVHD) | 1 |
| Eosinophilia | 1 |
| Gastroesophageal (GE) Junction Cancer | 1 |
| Esophageal Cancer | 1 |
| Duodenal Cancer | 1 |
| Cervical Cancer | 1 |
| Transitional Cell Cancer | 1 |
| Pancreatic Cancer | 1 |
| Bladder Cancer | 1 |
| Aplastic Anemia | 1 |
| Synovial Sarcoma | 1 |
| Angiosarcoma | 1 |
| Alveolar Soft Part Sarcoma (ASPS) | 1 |
| **Total** | **151** |

Supplementary Table 2. Distribution of disease etiologies for the TCIA Mediastinal LNQ dataset.

| **Clinical Indication** | **Count** |
| --- | --- |
| Lung Cancer (Non-Small) | 21 |
| Chronic Lymphocytic Leukemia (CLL) | 17 |
| Breast Cancer | 9 |
| Hodgkin`s Lymphoma | 9 |
| Lung Small Cancer | 8 |
| Adenocarcinoma – Unknown | 7 |
| Renal Cell Cancer | 7 |
| Thyroid Cancer | 6 |
| Endometrial Adenocarcinoma | 5 |
| Prostate Cancer | 4 |
| Neuroendocrine Cancer | 4 |
| Head and Neck Cancer | 3 |
| Esophageal Cancer | 3 |
| Rectal Cancer | 2 |
| Melanoma | 2 |
| Non-Hodgkin's Lymphoma | 2 |
| Mesothelioma | 1 |
| Cholangiocarcinoma | 1 |
| Ovarian Cancer | 1 |
| Diffuse Large B-cell Lymphoma | 1 |
| Esophageal Adenocarcinoma | 1 |
| Follicular Lymphoma | 1 |
| Angiosarcoma | 1 |
| Colon Cancer | 1 |
| Uterine Cancer | 1 |
| Leiomyosarcoma | 1 |
| Cervical Cancer | 1 |
| **Total** | **120** |

Supplementary Figure 1. Graphs plotting the detection metrics (precision, sensitivity, and F1-score) at different dose acquisition levels for the NoAugmentation model. Dotted curve represents the best fit.
